# Supplementary material for: Chromothripsis during telomere crisis is independent of NHEJ, and consistent with a replicative origin
Source: Genome Res. 2019 May;29(5):737–49. doi: 10.1101/gr.240705.118 (PMC6499312; doi:10.1101/gr.240705.118)
Supplement: Supplemental Material [file supp_gr.240705.118_Supplemental_file_1.zip › contigs/annotated_contigs/DB40/contig.5.DB40_length_632_mean_cov_9.21518987342.docx]

**DB40_length_632_mean_cov_9.21518987342**

```
AGTGAAGCTGTGTTCTCATCAGCAGAGACTCGCAGAGCATGGCGAGGGCGCAGCTGCGTTCTTCTGTGCACAGATGTCTGGAGTACCGC
 >chr18:10632-10919 - E=7e-134
GGAGGCGGAGCAGCGTTCTCCTCAGCACAGACCCGGGCGGGCGGGCAGTCCAGGGGCACCCCGAAGGTAGAGCTGTGTCCTCATCAGGA

CAGACCTTGCGGGCATTACCTCGCTTTGGGACAACTCGGGGCCAAAGCGACGGTAAATAAAATTCTTCCTCTTTGCTGCCCTGAACAAT

CAGGGTCAGAGTCCAGTTA|G|GGTTAGGGTCGGGGTCGGGGTCGGGGTCGGGGT|TAGGGGTTAGGGGTTAGGGTTAGGGGTTAG|G|
                    >chr10:135524558-135524592 + E=1e-08 p=0e+00   >chr12:133817862-1
GCAATTTGTGACATGTTTAGGAG|CA|GTAATGAATCCCTTATCTAGCCTGATGATTTTGCCGAGTAATGGGTAAAAGAAAGTAAAGTG
33817893 + E=6e-04 >chr11:125648384-125648410 - E=6e-04  >chr11:125648205-125648463 +
GATCTTAGCCTCCTTACTCTAAGAAATGGACAAAACAGGTCTGGCAGGAGGCGAAACCCCTAGGATTCTATAATAAGAATGGATTACAG
E=2e-140
GCCTCTCTGAAGATGAAGGAAAGGTTTGCTCCTAAACATGTCACAAATTGCCTGTTTACCAAGGACTTCAGAGAAGCCATGATTGTACC

TTTTATAGGGTCATGA
```
